# The Thermal Effects of Water Immersion on Health Outcomes: An Integrative Review

**DOI:** 10.3390/ijerph16071280

**Published:** 2019-04-10

**Authors:** Jiyeon An, Insook Lee, Yunjeong Yi

**Affiliations:** 1 Department of Nursing, Kyung-In Women’s University, Incheon 21041, Korea; jyan030@kiwu.ac.kr; 2 College of Nursing, Seoul National University, Seoul 03080, Korea; lisook@snu.ac.kr

**Keywords:** hydrotherapy, health promotion, water immersion

## Abstract

Hydrotherapy is widely used for the treatment and rehabilitation of patients, but it can also be applied to prevent diseases in healthy people. This review investigates the health effects of water immersion, a form of hydrotherapy, and the mechanisms by which the properties of water elicit such an effect. We searched PubMed, EMBASE, Cochrane Library, and CINAHL to identify relevant articles, of which 13 met the inclusion criteria. Various factors of water immersion were investigated in the 13 selected articles, including water temperature, immersion height, and application area. With respect to health effects, warm and cold water immersion affects the cardiovascular and neuromuscular systems, respectively. Nine articles focused on the effects of warm water immersion, explaining its thermal effect in relation to changes in disease-related serum substance levels and hemodynamic changes. While the sample population in most studies comprised young adults, two articles applied partial water immersion to the legs of elderly subjects to assess its effect on sleep. Because the water immersion protocols applied in the 13 articles were inconsistent, the health benefits could not be clearly explained. However, we expect the present findings to be beneficial for providing research guidelines for studies on the application of water immersion.

## 1. Introduction

Hydrotherapy is a field that pursues disease treatment or health effects using various properties of water for therapeutic purposes and is used synonymously with water therapy, aquatic therapy, pool therapy, and balneotherapy (hot spring and spa) [1]. The types of hydrotherapy are typically classified according to the various states of the water (liquid, solid, gas, or mixed state), but can also be classified according to the mechanical stimulation used, such as whirlpool, or the materials mixed with the water, such as mud [2]. Hydrotherapy is a therapeutic modality that maximizes the characteristics and advantages of water and is considered in clinical and alternative medicine to have an excellent therapeutic effect, with few adverse effects [3]. Water offers various advantages, including being abundant; not physiologically irritating; and having an excellent solvency, excellent viscosity, high heat capacity, and high heat conductivity. In addition, the density of pure water is similar to the average density of the water present in the human body, although it varies slightly, depending on body parts or temperature changes.

The health effects of hydrotherapy generally appear as thermal, mechanical, and chemical effects, either alone or as mixed effects. Thermal effects are elicited via heat (35–40 °C), body temperature (32–34 °C), or cold (8–10 °C) therapy. Heat therapy is typically explained by vasodilation and blood flow facilitation effects, while cold therapy is typically explained by vasoconstriction and pain reduction effects. Mechanical effects can be explained by the properties of water, such as buoyancy, hydrostatic pressure, and resistance, where the effect primarily appears when hydrotherapy is provided via immersion therapy. Buoyancy represents the force that opposes gravity, and when the body is partially or fully immersed, pain reduction and improvement in exercise ability occur due to the reduction of stress or application of weight to specific body parts. Hydrostatic pressure promotes blood flow by varying the pressure exerted on the body according to the immersion depth, which results in increased blood flow to major organs (the heart, brain, and lungs) or the promotion of diuretic action [4]. Resistance is the force that opposes bodily movement and is associated with the viscosity of the water and results in muscle strengthening. Chemical effects result from minerals, drugs, ions, oxygen, mud, and herbs added to pure water, which triggers chemical reactions on the skin to improve skin integrity and immunity [5].

Moreover, when hydrotherapy is conducted in ocean or mountain areas, the environmental effects can further enhance the psychological effects. Combining complimentary alternative therapies, such as massage, relaxation, music, or aromatherapies, can also induce health effects by increasing the body’s natural healing ability [6]. Exercise therapies or physical activities in water, including swimming, walking, and aerobics, are also combined for the purpose of physical therapy.

Hydrotherapy has been applied in combination with various therapies, such as physical therapy, rehabilitation therapy, disease treatment, and health promotion programs. Systematic reviews, review studies, and meta-analyses have been conducted on studies related to the application of hydrotherapy for symptom alleviation in patients with musculoskeletal diseases (such as arthritis and fibromyalgia), functional improvement in patients with neurological disorders (such as stroke and muscle paralysis), and rehabilitation of patients with acute injury in sports medicine [7,8,9,10]. Child birth-related hydrotherapy is also a popular form of hydrotherapy in clinical practice. In addition, recent studies have assessed the psychological effects of hydrotherapy, such as mental relaxation, mental fatigue, quality of life, and depression/stress [11,12,13]. With the large number of studies on the psychological and physiological effects of hydrotherapy, the need to expand the application of hydrotherapy and establish practical guidelines for the various clinical applications has been highlighted [14]. However, because hydrotherapy is limited by infrastructure and cost, its broader use is restricted. Nevertheless, if hydrotherapy has a positive effect on health promotion, as well as on disease prevention, treatment, and rehabilitation, then its application as an effective health promotion program can be expected.

In the field of health, hydrotherapy is commonly applied as local cold or heat therapy, and in particular, its effectiveness in reducing musculoskeletal symptoms and promoting post-traumatic recovery has been confirmed [15,16]. However, with water immersion, involving partial or complete immersion of the body, its effect has not been clearly explained due to the difficulties in application and limitations associated with possible adverse effects, the cost burden, and the physical environment. Nevertheless, the advantages of water are very diverse and effective in the field of health. There is evidence for the application of hydrotherapy for people with disease, but there is no such evidence for healthy people. Hydrotherapy for healthy people can be used as a health promotion program in the community and may be used as a new health service to achieve therapeutic effects beyond the scope of alternative medicine.

Accordingly, the present study aimed to conduct an integrative literature review to investigate the effective mechanism of water immersion to determine the effect of hydrotherapy using only the properties of water.

## 2. Materials and Methods

The present study was conducted in five stages, in accordance with the guidelines established by Whittemore and Knafl [17]. The first stage was the problem identification stage, in which the study objectives were set according to the need for a systematic review of the use of hydrotherapy in the field of health, and inclusion and exclusion criteria were clearly established for the articles to be analyzed. The second stage was the literature search stage, in which search engines and search terms that fit the study objectives were selected and appropriate search strategies were planned. Generally, an integrative review process aims to minimize various biases by including as many primary sources as possible, but the present study planned to apply a consistent search strategy via search formulas to increase the rigor of the studies being analyzed. The third stage was the data evaluation stage, in which the study objectives, design, methods, and populations in the articles identified in the search were thoroughly evaluated to extract the articles to be included in the final analysis. The fourth stage was the data analysis stage, in which the articles to be analyzed were arranged, codified, categorized, and summarized to derive a comprehensive conclusion. This stage consisted of a detailed analysis of the application of hydrotherapy—by which method, for which subjects, and for what objective—and what the meaningful findings were in the studies. The fifth stage was the presentation stage, in which the major contents of the analyzed articles were graphically depicted or described according to the search objectives.

### 2.1. Problem Identification Stage

The objective of the study was to perform an integrative review of the effects of water immersion on physical or psychological health effects among the general population. Water immersion can be defined in a variety of ways. We defined the immersion of some part or all of the body (submerged form) in water (liquid state) as water immersion. We limited this study to wet hydrotherapy in which the body part made direct contact with heated or cooled water. The detailed inclusion and exclusion criteria applied to the articles to be analyzed for this objective were as follows:

#### 2.1.1. Inclusion Criteria

- Articles published outside Korea between January 2008 and October 2018
- Articles with application of hydrotherapy in the form of water immersion using pure water (liquid state)
- Journal articles published after review by the editorial board
- Published articles in full text form, including the abstract, and written in English
- Research articles excluding systematic or literature reviews

#### 2.1.2. Exclusion Criteria

- Articles with application of hydrotherapy other than water immersion
- Articles with application of mechanical stimulation using water (whirlpool, etc.)
- Articles with application of hydrotherapy combined with aquatic exercise
- Articles with application of hydrotherapy combined with physical therapy
- Articles with application of hydrotherapy for patients with specific diagnoses
- Articles with application of hydrotherapy that included hot springs or mixtures (oil, mud, sulfur, salt, minerals, etc.)
- Articles with hydrotherapy applied as part of a multimodal intervention
- Articles with application of cooling therapy as hydrotherapy after artificially inducing high body temperature
- Articles with application of hydrotherapy for the purpose of recovery after exercise and/or physical activity
- Articles with hydrotherapy applied for sports athletes or specific occupational groups (firefighters, divers, etc.)
- Articles with a research objective that did not match that of the present study or articles with full text that could not be accessed

### 2.2. Literature Search Stage

The selection of search terms and construction of search formulas for the literature search were performed between 15–19 September, 2018, while the literature search was performed between 10–15 October 2018 by concurrently searching using four search engines. The results of the preliminary search confirmed the existence of a systematic review that analyzed the effects of water immersion in patients with specific diseases, but no existence of reviews that analyzed the effects of water immersion in the general population.

**Figure 1:**
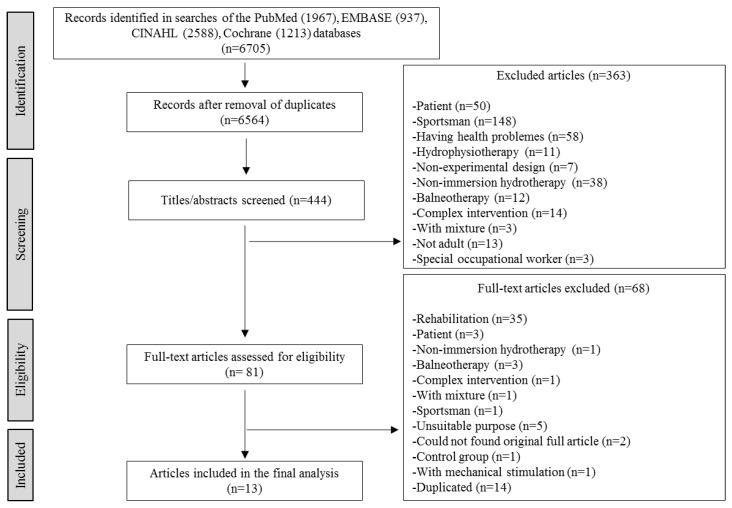
Flowchart for processing.

The search terms and formulas were specified through PICO construction. Because the objective of the present study was to conduct an integrative review on the application of water immersion in certain populations, no limitations were set in terms of the study population. To include all forms of hydrotherapy as interventions, the search terms used both Medical Subject Headings (MeSH) terms and text words. “Immersion,” “bath,” “hydrotherapy,” and “balneotherapy” were used as MeSH terms, while “bathing,” “hot-water immersion,” “cold-water immersion,” “heating,” and “cooling” were used as text words. Separate search terms were not used for comparisons; MeSH words and text words were also used for outcomes. “Physiology,” “psychology,” “health promotion,” and “mental health” were used as MeSH terms, while “wellbeing,” “wellness,” “healing,” and “meditation” were used as text words. Among the major international medical databases, PubMed, EMBASE, Cochrane library, and CINAHL were searched. EMBASE and CINAHL, which do not use MeSH indexing, were searched by substituting the terms with those used in the EMTREE and CINAHL headings in the corresponding databases. To establish the search strategies, advice from search experts was received and searches were performed after confirming database access from the library of the organization the researcher is affiliated with. To increase the sensitivity of the extracted articles and integrative article extraction, unlike systematic reviews, pure experimental, quasi-experimental, and pre-experimental designs were all included. The search returned 1967, 937, 2588, and 1213 articles from PubMed, EMBASE, CINAHL, and Cochrane library, respectively. After removing a total of 141 duplicate articles, the remaining articles were assessed based on the inclusion and exclusion criteria. Consequently, a total of 13 articles that passed the quality assessment were ultimately included in the present study (Figure 1).

### 2.3. Data Evaluation Stage

Evaluation of the quality of the articles was based on the inclusion and exclusion criteria, in which the criteria were strictly applied through in-depth discussions among the researchers. Reference management software was used to thoroughly review the reference information in each article, and a rating score was assigned to indicate the relevance of each article, while article extraction was performed according to rating score-matching among the researchers. Because the meaning and definition of hydrotherapy varied among studies, discussion of the assessment of the quality of the articles was also added to the final article extraction process, and since there were no other updated articles or articles found by manual searching based on the inclusion and exclusion criteria, no additional articles were included.

### 2.4. Data Analysis Stage

Data integration of the final articles selected for analysis was based on agreement among the entire research team. For the data analysis, the researchers attended weekly meetings to discuss the methods for presenting the findings. As a result, the findings presented in Table 1 and Table 2 were derived.

### 2.5. Presentation

Based on the integrative review of the 13 articles included in the final analysis, the major findings on the health effects of hydrotherapy on healthy subjects are presented in Table 1 and Table 2. 

## 3. Results

### 3.1. Study Design/Time for the Measurement/Comparative Group

Among the 13 articles analyzed, one group pre- and post-test designs and randomized crossover designs appeared in four articles each. The studies with randomized crossover designs applied two or more experimental treatments over a certain period to the same subjects, and thus, the present study conducted comparisons with the self-control group (placebo or control) when analyzing the effects of water immersion. In addition, there were two randomized controlled trials, including one controlled single-blinded parallel trial, and three studies of quasi-experimental designs. 

The number of measurements used to test the effects of dependent variables ranged from two to six. The study by Brunt et al. [25] had the highest number of measurements (six), including one pre-test and five post-test measurements. Even in cases with a single experimental treatment, repeated (two or four) measurements were performed to investigate the short- and long-term effects of water immersion.

In studies that used designs other than a one group pre- and post-test, the types of treatment applied to the control group included using different temperatures to emphasize the thermal effect or testing the physiological effect via comparison with sedentary seating or cycling.

### 3.2. Intervention

Among the 13 articles that divided water immersion into two categories (warm and cold) for an investigation of the thermal effect, nine used warm water immersion, three used cold water immersion, and one used both warm and cold water immersion. When warm water immersion was applied, the temperature range was 38–48 °C, and the immersion was subcategorized as warm water immersion (41 °C, 42 °C, and 41–43 °C) or hot warm immersion (38 °C, 40 °C, 42 °C, 41–42 °C, 47–48 °C, and 48 °C), depending on the temperature. However, it was difficult to differentiate between warm and hot warm immersion by temperature alone. In contrast, cold immersion was conducted using a temperature range of 3–26 °C, but the temperature was maintained below 15 °C in most articles. Only one article maintained the temperature at 26 °C and referred to this as mild cold water immersion. 

The immersion methods varied and included the use of a bathtub, plastic bucket, tank, acrylic container, chamber, and bath machine. Surprisingly, no articles applied hydrotherapy in a wide space, such as a swimming pool. With respect to the immersion position, the subjects were immersed while sitting in a chair in most articles, but in the study by Shimodozono et al. [19], the subjects were lying on a stretcher tilted at a 36° angle for whole-body immersion.

Immersion height relative to parts of the body varied, with four articles using full immersion with a height of at least up to the xiphoid process, one article using half immersion up to the navel, and eight articles using partial immersion with only a part of the body immersed. Among the articles using partial immersion, all articles applied immersion on the extremities, with five articles applying it on the lower extremities and three articles applying it on the upper extremities.

Most of the articles that considered the risks associated with the thermal effect of hydrotherapy measured the core temperature of the subjects during hydrotherapy, primarily via the rectum. Moreover, a rest period was included as a pre-treatment measure to reduce the risks associated with sudden temperature change. In addition, after hydrotherapy, clothing or blankets were used to maintain the body temperature, and the experiment was completed after allowing the subjects to rest sufficiently at room temperature, resetting the body temperature to normal.

To investigate the physiological changes after hydrotherapy, each article included confounding factors in the exclusion criteria to place limitations on the subject selection process. Subjects with specific health issues were excluded in 11 of the 13 articles, wherein cardiovascular disease was the most common health issue. Because the articles were designed to assess the thermal effect, which may not necessarily involve a health issue, patients with heat-related illness, hot or cold adverse reactions, and cold-induced illnesses were also included in the exclusion criteria [18,22,24].

In the studies by Shimodozono et al. [19], Bailey et al. [20], and Hu et al. [23], female subjects were allowed to participate in the experiment during the follicular phase of their menstrual cycle in order to control for the temperature changes due to the menstrual cycle, while middle-aged women were only enrolled if they were in the menopausal stage. In a study by Brunt et al. [25], urinalysis was performed on female subjects during all experimental procedures to accurately identify their pregnancy status by assessing the level of human chorionic gonadotropin, a pregnancy hormone. In addition, other lifestyle factors, such as medication use, drinking status, smoking status, exercise status, caffeinated beverage intake, estrogen hormone therapy, and regular thermal therapy, were assessed in order to select a homogenous study population and control for the confounding effects on the treatment effect.

In most of the articles, pre-treatment was applied prior to the experiment, which varied depending on the measurement variables and experiment type. For example, in the study by Shimodozono et al. [19], because a serum test was part of the post-test, the subjects were instructed to fast for 12 h prior to the experiment and only drink 300 ml of water on the day of the experiment. In the studies by Blunt et al. [18,25], urine samples were analyzed to ensure that the first morning urine on the day of the experiment had a specific gravity ≤ 1.024 to reduce the risks associated with changes in the volume and composition of body fluids due to hydrotherapy. Subjects with a specific gravity of urine > 1.024 were instructed to drink 5 ml of water per kg body weight prior to immersion. In four other articles, vigorous exercise and intake of alcohol, caffeine, food, and beverages were restricted prior to participation in the experiment [20,22,23,28], while the studies by Herrera et al. [22] and Wakabayashi et al. [24] required participation in the experiment at set times in order to minimize the circadian effects that can influence changes in body temperature during the day.

A total of seven articles mentioned the application of post-hoc treatment immediately after the experimental treatment, including the immediate removal of moisture after moving to an environment prepared for recovery from changes in body temperature (recovery chair, laboratory, and warm room). In the two studies by Brunt et al. [18,25], the subjects were monitored until their rectal temperature reached 38.5 °C, while in the study by Herrera et al. [22] that applied cold water immersion, the subjects were instructed not to apply friction to the area where the immersion was applied, in order to minimize tissue damage after the experimental treatment.

### 3.3. Participants

In all 13 articles, the participants consisted of healthy adults of a broad age range. Only two of the 13 articles included elderly participants, while ten articles included adults aged 20–39 years. The study by Hu et al. [23] divided the participants into healthy young and elderly women to compare the effects of hydrotherapy according to age. In five articles, the participants consisted of similar proportions of men and women, while five other articles only included study participants of one sex. The five articles calculated the sample size when determining the number of participants [18,22,26,29,30]. One article was a randomized controlled trial, but there was no sample calculation procedure taking into account statistical power and effect sizes [20].

### 3.4. Time Per Session/Total Session or Period

The time per session applied for experimental treatment ranged from 10 to 90 min. In eight of the 13 articles, only a single session was applied. The total number of sessions ranged from one to 42, and most of the articles involving ≥ 2 sessions applied partial water immersion. The studies by Bailey et al. [20] and Brunt et al. [25] had the longest experimental treatment period, which was eight weeks.

### 3.5. Outcome Variables

The outcome variables included the status of the vascular, hematological, cardiorespiratory, neuromuscular, and thermoregulatory systems; cognition; sleep behavior; and pain control, which can be largely categorized into physiological parameters and cognitive behavioral parameters (including sleep behavior). Most of the articles discussed physiological parameters; however, three articles measured cognitive function and sleep problems as effect variables of water immersion. Among the physiological parameters, vessel function (e.g., dilatation, stiffness, and thickness) and blood flow were the most common, while the other included variables primarily consisted of those associated with cardiopulmonary function (e.g., blood pressure, respiratory rate, and heart rate), substances in the blood (e.g., oxy-hemoglobin, lipid, glucose, and blood cell count), hormones (insulin, cortisol, adiponectin, leptin, and neurotrophic factor), and pain mechanisms (nerve conduction parameters and pain intolerance). With regard to the cognitive behavioral parameters, two articles [29,30] included measurement variables for quantity and quality of sleep.

### 3.6. Conclusion and Implementation of Evidence

The most apparent conclusions with regard to the physiological effects of warm water immersion were that it caused flow-mediated dilation of the artery to prevent ischemia-induced vascular dysfunction, or exhibited an occlusive reactive hyperemia effect, whereby it elicited the same effect as exercise to improve cardiopulmonary function [18,20,23,25,27]. These hemodynamic effects of warm water immersion were explained in each study as follows. Bailey et al. [20] compared the exercise group with a control group to estimate cardio-respiratory function (maximal oxygen consumption) during water immersion. Surprisingly, cardio-respiratory fitness in the warm water immersion group was similar to that of the exercise group on land. Hu et al. [23] also explained that improvements in arterial stiffness due to an elevated core body temperature could also improve cardiovascular function by decreasing vascular resistance and increasing blood flow. 

The increased diameter of the artery caused by warm water immersion reduces shear stress, which is a force acting against a blood vessel wall, directly proportional to the blood flow velocity and inversely proportional to the diameter of the blood vessel. Increasing the diameter of the artery and deceasing shear stress may minimize the endothelial damage of the blood vessels and may reduce pathological changes such as arteriosclerosis, thus improving the blood flow [18,20,23,25]. Moreover, flow-mediated dilation of the carotid artery reduced the arterial wall thickness or increased the blood pressure to improve the vessel endothelium function [23,25]. Pathological changes such as atherosclerosis, which thicken the arterial wall, cause structural changes in the vascular endothelium, inflammatory reactions, and hemodynamic stimulation by changing shear stress and blood pressure. Bunt et al. [18] explained that warm water immersion may reduce the progress of vascular pathologic changes, such as atherosclerosis. Furthermore, warm water immersion increased oxy-hemoglobin levels to improve tissue oxygenation, and also contributed to an improved short-term brain function by increasing the levels of substances involved in brain-cell genesis. Wijayanto et al. [27] explained that the improvement of blood flow rate by warm water immersion therapy can, in turn, improve the function of important organs such as the brain by facilitating the transport of substances in the blood. In other articles, partial immersion applied to elderly subjects was found to be effective in partially improving the quantity and quality of sleep [19,29,30]. In conclusion, the physiological changes induced by warm water immersion, such as vasodilation, increased blood flow, reduction of arterial stiffness, vascular endothelial function, oxygenation, and decreased sleep-related stress, may result in improvements in the cardiovascular function. These physiological changes due to water immersion are similar to the cardiovascular effects of physical activity. Exercise has a variety of effects, particularly the improvement of cardiovascular function, increased cardiac output, decreased atherosclerotic plaque formation, decreased vascular resistance, increased organ perfusion, improved insulin-sensitivity, increased oxygen carrying capacity, and improved plasma lipid profile [31]. 

In contrast, cold water immersion reduced the nerve conduction velocity, which raised the pain threshold to promote pain control. Moreover, it also increased the blood pressure and heart rate variability, the latter of which is an indicator of sympathetic activity. One article used repeated cold water immersion and identified 26 °C as the appropriate temperature for insulative cold adaptation [24].

Assessment of vascular status and hemodynamic changes as a thermal effect of water immersion was an important part of the methods applied in the included studies. Most of the articles used direct measurement methods using medical equipment because physiological indicators were used as dependent variables. For example, the diameter of the arterial blood vessels was directly measured using a medical device such as an ultrasound machine or Doppler flowmetry [18,20,24,25]. The stiffness of the vessels was measured using a vascular screening system (CAVI-VaSera VS-1000; Fukuda Denshi, Tokyo, Japan) [23].

## 4. Discussion

The findings of this integrative review of the health effects of water immersion can be summarized as follows: the thermal effect of water immersion was tested in all 13 articles; water immersion was applied for healthy adults in various forms, according to immersion height (full vs. partial immersion) and water temperature (warm vs. cold immersion); and the intervention effects were primarily assessed by measuring physiological parameters. The combined results for water immersion can be used as evidence to establish guidelines or recommendations for health programs or services. We expect water immersion to be utilized as a cost-effective and highly-valued therapeutic modality, assuming that its positive health effects can further promote health among healthy people.

The ultimate goal of the present study was to investigate the mechanisms by which the unique properties of water (i.e., thermal conductivity, buoyancy, hydrostatic pressure, and resistance) elicit the water immersion effect. However, most of the included articles mentioned the effects with an emphasis on thermal conductivity, and although the effects of buoyancy and hydrostatic pressure were expected in full immersion, none of the articles provided separate explanations for such effects. The studies by Brunt et al. [18] and Shimodozono et al. [19] described one of the limitations as being unable to differentiate mechanisms based on the thermal effect and hydrostatic pressure with respect to changes in the serum adipokine level, which was one of the dependent variables. Because water itself has various properties, it cannot be confirmed that the effect of the intervention performed using water was due to a single property. Meanwhile, another study reported that although warm water, heated to body temperature, would be expected to have little thermal effect, continued friction generated by the warm water contacting the skin improved the blood flow throughout the body. As a result, the heart rate decreased to minimize the heart burden due to the increased direct brachial artery blood flow to the cardiac vessels and the blood volume returning to the heart [32]. 

In contrast, a study by Brunt et al. [25] implemented water immersion in the sitting position in order to only identify the thermal effect while minimizing the effect of hydrostatic pressure. When the water level is higher than the xiphoid process, a fluid-shifting effect appears wherein the peripheral blood moves to the center, resulting in an increased cardiac output and decreased heart rate [5]. Brunt et al. [25] explained that because their study aimed to examine changes in vasodilation and hyperemia due to the thermal effect of warm water immersion, using a sitting position was the most appropriate method for minimizing the fluid-shift effect in order to control hyperemia due to hydrostatic pressure. Among the five studies included in the present study that examined water submersion above the waist, four studies were conducted in a sitting position. 

In this study, the mechanism of thermal vasodilation due to warm water immersion results in hemodynamic improvement. Tei et al. [33] reported that such hemodynamic improvement reduced the cardiac preload and afterload in patients with congestive heart failure, indicating its clinical significance as a non-pharmacological therapy. The thermal effect of warm water immersion can be considered an effect of the warm temperature of the water, but in addition to water immersion that involves placing the body in water (liquid state), the same effect may be exerted using various heat delivery methods. In addition to the method of heating using liquid, such as water immersion, heating using non-liquid states, including sauna therapy (e.g., Finnish stream, radiant-heat, and dry-heat sauna), can induce various physiological responses to improve clinical symptoms, such as pulmonary congestion, myocardial infarction, hypertension, and congestive heart failure, as well as aid the reduction of depression and chronic fatigue [34]. In the present study, we only analyzed the effect of an immersion bath, which involves direct contact with water (liquid state). Therefore, we suggest that further analysis of the effect of an immersion bath using non-liquid state-based heating is required.

In particular, the thermal effect of water immersion may be even larger since it can be applied via the delivery methods of both convection and conduction. Among the articles analyzed, the study by Herrera et al. [22] compared the neuromuscular effects of water immersion among three different cold modalities (cold water immersion, ice massage, and ice pack), in which measurement of the calf nerve conduction velocity indicated that cold water immersion had the largest effect. Two mechanisms underlying the greater effect of cold water immersion were suggested. First, because the body surface area exposed to cold temperatures is larger for cold water immersion than for other modalities, the cooling effect acted on not only the superficial nerves, but also on the subcutaneous tissues. Second, both ice massage and ice packs have singular effects due to conduction, but cold water immersion had a dual effect due to conduction and convection. 

Achieving physiological effects by cooling only a specific area of the body, as in the study by Herrera et al. [22], is defined as cryotherapy. Among the articles analyzed, the study by Costello et al. [21], which also applied cold water immersion as a form of cryotherapy, investigated whether partial cold water immersion can reduce knee joint position sense. Joint position sense is the sense needed to maintain proper joint function by subconsciously recognizing joint position, but cooling is known to induce the negative effect of reducing joint position sense by reducing the nerve conduction velocity [35]. However, the study by Costello et al. [21] demonstrated that cold water immersion did not reduce the joint position sense and established an optimal temperature of 14 ± 1 °C and optimal time of 30 min for the cryotherapy protocol. Two studies found that cryotherapy does not reduce joint position sense [36,37]. Khanmohammadi et al. [36] suggested that cryotherapy is not deleterious and can be safely used without fear of reinjury due to decreased proprioception. 

Nevertheless, two articles showed that cold water immersion has a particularly large effect on the neuromuscular system [22,38]. Besides reduction of the nerve conduction velocity, cooling also has an anesthetic effect due to the increased threshold of subcutaneous sensory receptors. Algafly et al. [38] showed that cooling a body part reduces the nerve conduction velocity and inhibits nociceptive receptor sensitivity, thereby relieving pain. Cryotherapy and cold therapy using cold water immersion can minimize pain while allowing movement or exercise, and thus, can be used for the musculoskeletal function of healthy people, as well as rehabilitation for patients with specific diseases [39].

One of the findings of the present analysis was that there are no current standard guidelines for water immersion. The intervention protocols were not consistent across studies, and the time per session and total period of experimental treatments also varied. Despite the presence of risk factors that can cause sudden changes in body temperature, information on safety precautions was lacking. Many exclusion criteria may have been used in the 13 articles analyzed for the purpose of minimizing the confounding effects on the dependent variables. This may also have been a strategy to limit the study population to those individuals who do not have health vulnerabilities in order to prevent the occurrence of adverse effects after water immersion.

When the safety considerations in the 13 articles were assessed to analyze the health effects while minimizing the adverse effects of water immersion, they could be divided into pre- and post-treatment precautions, although detailed descriptions were not available. Although cold or hot water immersion was applied for a long period of time, there was a lack of sufficient consideration of the safety of the participants [26,27]. Otherwise, in the studies by Brunt et al. [18,25], the urine specific gravity of each subject was measured as a pre-intervention preparation, and if there were any concerns regarding fluid deficit or dehydration based on measured values exceeding 1.024, the subject was instructed to drink 5 mL/kg of water before participating in the experiment. Due to water immersion, atrial natriuretic factor is secreted from the atrium, which expands due to hyperemia caused by blood flow to the center of the body, and this substance may cause fluid deficit due to inhibition of the renin-angiotensin-aldosterone system, diuresis, and Na^+^ excretion [3,4]. Another study reported that diuresis due to water immersion can increase further at lower temperatures during cold water immersion [40]. Thus, water immersion is associated with the risk of loss of body fluid; therefore, precautions regarding monitoring of hydration before and after experimental treatment, and homeostasis of body fluids are essential. Jimenez et al. [4] warned that if the whole body was immersed for long periods of time (over six hours), health problems such as hypovolemia due to the loss of water and sodium could occur. Brunt et al. [25] assessed the whole body sweat rate and body fluid homeostasis after the experimental treatment. For the above reasons, therapists using water immersion should monitor the risk of body fluid loss of the participants, and if the participant is a child or elderly individual with vulnerability to body fluid loss, the therapist should be prepared for an emergency situation.

Water immersion is associated with the risk of changes in body temperature according to the water temperature. Therefore, it is necessary to monitor the patient for signs of hyperthermia and hypothermia by measuring the body temperature before, during, and after the intervention. Among the articles analyzed, the studies by Brunt et al. [18,25] measured rectal temperature, representing the core temperature, before and after the experimental treatment, in order to monitor the changes in body temperature. The studies by Bailey et al. [20] and Herrera et al. [22] were also designed to maintain body temperature in spaces with the temperature set to 21 °C and 24 °C, respectively. Other articles also contained explanations of precautions for removing moisture or keeping warm immediately after immersion. However, only two of the 13 studies determined whether the core body temperature returned to normal, while the remaining studies did not provide separate explanations for the protocol for determining whether the body temperature had recovered. When the body temperature drops below 32.2 °C after cold water immersion, adverse events, such as dysrhythmias, a decreased level of consciousness, a decreased respiratory rate, hyporeflexia, and hypotension, may occur [41], and after hot water immersion, hyperthermia may cause health issues, such as heat stroke and severe burns [42]. Therefore, when applying water immersion, it is necessary to monitor the ability to control body temperature prior to the experimental treatment and ensure the recovery of body temperature after the experimental treatment. In particular, cold water immersion may have greater perceived physical discomfort and physical adverse effects than warm water immersion. Thus, water immersion may have side effects such as skin maceration, skin softening, edema, hyperthermia or hypothermia, and excessive vasodilatation or vasoconstriction [5,43]. Especially, hot water immersion of 45 to 50 °C or more may cause damage to cells due to protein denaturation and the sudden immersion into cold water may cause vasoconstriction [5]. Therefore, in order to ensure safety during water immersion, it is important to determine the possible side effects by monitoring physical indicators and subjective discomfort. 

Moreover, in the articles analyzed, assessments of alcohol, caffeine, and chocolate intake; exercise; and bathing were included in the pre-intervention preparation. The reason for this was that the vasomotor effects of these factors may influence the vasomotor effect due to water immersion. Because these factors also affect sympathetic activity, they may also influence the sympathetic effects of water immersion [44].

In some of the articles analyzed, restrictions were placed on female subjects with respect to their menstrual cycle and circadian effect to eliminate exogenous variables that may influence changes in body temperature, aside from the thermal effect due to water immersion. In particular, estrogen plays a very important role in regulating body temperature in women [45]. One study that enrolled young female participants only included women who were in the follicular phase of their menstrual cycle [23]. In another study that enrolled older women, participation was limited to those in the post-menopausal phase, because significant fluctuations in body temperature occur during menopause [46]. Moreover, the human body temperature is known to follow a certain cycle in the circadian rhythm, wherein the body temperature is lowest in the early morning and highest in the early evening, with a typical difference of 0.9 °C [46]. It was found that applying the experimental treatment in the morning had less influence on the thermal effect of water immersion than application of the treatment in the afternoon. However, two of the included articles implemented the experimental treatment at the same time every day in consideration of circadian effects; the study by Herrera et al. [22] applied the treatment at the fixed time range of 14:00–18:00, and the study by Wakabayashi et al. [24] mentioned that the experimental treatment was implemented at the same time, but the exact time was not mentioned.

In addition, the thermal effect of water immersion caused changes in the concentration of substances in the blood (blood cells, hormones, lipids, etc.). In particular, the study by Shimodozono et al. [19] reported that warm water immersion played a direct role in preventing diseases by increasing the levels of adipocyte-derived hormones, which have been associated with obesity and inflammatory disorders. The study by Kojima et al. [28] reported that water immersion was beneficial in maintaining brain function and homeostasis by increasing the concentration of brain-derived neurotrophic factor, a neuron-inducing factor, and reducing the concentration of cortisol, which is commonly referred to as the stress hormone. Such changes in the concentration of substances in the blood can be explained by the direct effect of disease prevention and treatment. Thus, it is necessary to gather more evidence in future repeat studies.

## 5. Conclusions

Based on the findings of the present study, it was determined that water immersion applied to healthy adults can cause physiological and cognitive behavioral changes according to the thermal effect of water. For humans, maintaining body temperature represents an important homeostatic phenomenon; thus, the studies mostly included young adults with almost no health issues in order to minimize adverse effects due to sudden changes in body temperature caused by water immersion. As an exception, one study [30] that used the quantity and quality of sleep, among cognitive behavioral changes, as dependent variables, was conducted on elderly subjects. Because the human body temperature is affected by changes based on the circadian rhythm, hormonal changes, and sex-based physiological differences, the inclusion and exclusion criteria used to select the subjects were extensive. Moreover, a review of the pre-intervention preparation for eliminating confounding effects that can influence the dependent variables was essential, as was post-intervention management for the prevention of physiological adverse effects due to water immersion.

One of the noteworthy points regarding water immersion was that warm water immersion was effective for cardiovascular function improvement, and thus, has clinical significance as an alternative therapy to exercise training. The mechanism of improvement of cardiovascular function can be explained by the elevation in cardiac output due to the blood flow shift to the main blood vessels. Although the results were derived from only one study, cold water immersion reduced the nerve conduction velocity to help reduce pain, and elicited a positive effect on cardiovascular function via the modulation of vasomotor variability. Health promotion acts across all stages of wellness and disease. Water immersion contributes to improvements in health maintenance, from cardiovascular function to musculoskeletal pain relief. 

We expect to use the findings in the present study to establish standard guidelines that can explain the effects of water immersion. Although the results were presented in a variety of ways, it is difficult to suggest standard guidelines, but some considerations for water immersion applications are as follows. First, the therapist must check the temperature of the water and core body temperature of the participants to maintain body temperature homeostasis. Second, the therapist must carefully check the hydration status of participants to prevent excessive fluid loss during immersion, prepare a quick method of drying or warming after application, and limit the consumption of food and beverages that cause diuretic effects. Third, the therapists should consider the sudden hemodynamic changes and the skin damage, taking into account the negative aspects of water immersion. Finally, it may be advisable to not use unnecessary chemical additives because it may cause alterations in plasm osmotic pressure due to dissolved solutes in the water. In addition, a limitation of the analyzed studies, which in turn limits the interpretation of the present study findings, is that the thermal effect and other effects could not be explained separately and did not explain whether there was a confounding effect of negative aspects (e.g., skin irritations, personal vulnerability to water immersion, etc.) of water immersion on the outcome variables. Another limitation of the present study is that only the studies published in the past 10 years were analyzed. The type of water immersion actually applied, with regard to the water temperature, immersion height, immersion position, and physical environment for immersion, was inconsistent among studies. Most of the analyzed studies focused on the thermal effects of water, but in fact, they did not rule out the effects of hydrostatic pressure. Some studies stated that other effects of water were controlled for and that only thermal effects were examined. However, considering the multiple mechanisms of water, it is difficult to completely control for water actions (hydrostatic pressure, buoyancy, resistance, chemical effects). 

We recommend the following for future iterative studies investigating the health effects of water immersion: a sufficient review of exclusion criteria, the establishment of safety precautions for changes in body temperature, controlling for exogenous variables, and the establishment of protocols for water immersion.

## Figures and Tables

**Table 1:** Exclusion criteria and safety considerations in the included studies.

Author	Exclusion Criteria	Safety Considerations	
		Pre-Intervention Preparation	Post-Intervention Management
Brunt et al. [18]	• Health problems; cardiovascular disease, diabetes mellitus, hypertension, hyperlipidemia, recent surgery, dermatological conditions, and history of heat-related illness • All medication for 24 h, alcohol and caffeine intake for 12 h, and heavy exercise for 24 h prior to experiment • Pregnant woman	• If the first morning urine specific gravity was >1.024, subjects drank 5 mL/kg water • Rectal thermistors were used as a safety precaution	• Subjects were transferred to a recovery chair for at least 10 min, or until rectal temperature had fallen below 38.5 °C
Shimodozono et al. [19]	• Health problems and cardiovascular disease • All medication and supplements • Women; to avoid hormonal effect of the menstrual cycle	• Water immersion was performed after overnight fast • Subjects drank only 300 mL of water at 7 A.M.	• Subjects were kept warm and were wrapped in a blanket for 30 min
Bailey et al. [20]	• Health problems, cardiovascular and metabolic diseases • All medication (including hormonal contraceptives) and smoking • Women with irregular menstrual cycles (~28 days)	• Checking alcohol intake, exercise for 24 h and caffeine intake for 12 h prior to experiment	• Laboratory temperature was controlled (21 °C and 45% relative humidity)
Costello et al. [21]	• Health problems; Raynaud’s disease, ankle or knee injuries for 12 months prior to experiment, and history of ear or vestibular conditions	-	• Subjects wore clothes and were transferred to the laboratory
Herrera et al. [22]	• Health problems; peripheral vascular disease, cardiovascular disease, diabetes, neurological disorders, skeletal muscle disorders, recent trauma or injury to leg, local hot or cold insensitivity, cold adverse reactions, Raynaud’s phenomenon, and pregnancy	• Subjects attended intervention at the same time (e.g., 2–6 P.M.) • Checking alcohol, caffeine, or chocolate intake for 2 h, and exercise for 4 h prior to experiment	• Subjects were transferred to a warm room at 24 °C • Leg was quickly dried without friction
Hu et al. [23]	• Health problems and any disease known to affect the cardiovascular system • Smoking and regular physical activity • Medication for disease treatment; diabetes, metabolic disease, and cardiovascular disease • Estrogen-replacement therapy • Regular thermal therapy; sauna, bathing, and footbath • Young women not were scheduled in the follicular phase of their menstrual cycle • Older women before menopause	• Checking alcohol or caffeine intake, vigorous exercise, and bathing for 24 h prior to experiment	-
Wakabayashi et al. [24]	• Health problems and history of repeated cold exposure or cold-induced illness	• All measurements were conducted at the same time of the day to reduce circadian effects	-
Brunt et al. [25]	• Health problems and history of cardiovascular-related diseases • All medication (including hormonal contraceptives) • Pregnancy and using urine human chorionic gonadotropin (only women)	• Subjects were confirmed to be in a state of euhydration via urine-specific gravity (<1.02) • If the specific gravity was >1.02, subjects drank 5 mL/kg water	• Subjects were monitored for 10 min, or until rectal temperature had fallen below 38.5 °C • Dry nude body weight of subjects was measured to calculate the mean whole-body sweat rate, after correcting for water intake during immersion
Streff et al. [26]	• Health problems; any medical, neurological, psychiatric, or psychological disorders, and substance abuse (e.g., nicotine) • All medication (except oral contraceptives) • Alcohol intake for 24 h prior to experiment	-	-
Wijayanto et al. [27]	-	• Checking eating, intake of alcohol or caffeinated beverages, smoking, and exercise for at least 2 h prior to experiment	-
Kojima et al. [28]	• Medication for disease treatment and medical or psychological condition • Smoking and regular exercise	• Checking eating and drinking of any fluid except tap water	-
Valizadeh et al. [29]	• Health problems; enuresis, use the other complementary treatment except hypnotic drugs, and diabetes for more than 10 years	-	-
Kim et al. [30]	• Health problems; foot injuries, sensory disorder, acute disease, peripheral vascular disease, schizophrenia, pain or infection, and difficulties in communicating	-	• Subjects were examined for any redness or pain while completely drying feet • Subjects wore socks to keep their feet warm

**Table 2:** Result of review of the included studies.

Authors	Design	Experimental Group					Comparative Group	Outcome Variables	Conclusion and Implementation of Evidence	Effect of Water
		Intervention	Time (T) for Measurement	Participants	Time Per Session	Total Session and Period				
Brunt et al. [18]	One group pre- and post-test	• Hot warm water immersion of the shoulder (40.5 °C) for 25–30 min • Sitting up in waist-level water in a tub for 60 min	• Pre-treatment (T1) • Post-treatment (T2) • Post-I/R (T3)	• Young, healthy men (n = 5) and women (n = 5) • Aged 23 ± 6 years (mean) • Sample size was calculated using SigmaPlot 11.0	60 min	Single	-	• Brachial artery flow-mediated dilation as endothelial function • Rectal temperature • Heart rate	• There was a significant interaction effect of intervention × time point on FMD% • Rectal temperature increased to a peak of 38.9 ± 0.2 °C • Heart rate increased from 81 ± 18 beats/min at T1 to 127 ± 18 beats/min during hot warm immersion • Hot water immersion results in potential protective effects against I/R-induced vascular dysfunction	Thermal effect
Shimodozono et al. [19]	One group pre- and post-test	• Warm immersion to subclavicular level (41 °C) • Subjects were reclined on a stretcher at an angle of 36° in a bathtub	• Pre-treatment (T1) • Immediately post-treatment (T2) • 30 min after treatment (T3)	• Healthy men (n = 7) • Aged 39.7 ± 6 years (mean) • No mention of calculation of sample size	10 min	Single	-	• Adiponectin and leptin, as adipocyte-derived hormones • Glucose • Insulin • Lipids (T-chol, LDL-C, HDL-C, TG, and FFA) • CBC (RBC, Hb, Ht, WBC, and Plt)	• Leptin levels significantly increased at T2 and T3 after warm immersion • Some parameters (insulin, T-Chol, RBC, Hb, Ht, and WBC) significantly increased immediately (T2) after warm immersion • A single warm immersion for 10 min may modulate leptin and adiponectin profiles in healthy men	Thermal effect
Bailey et al. [20]	Randomized controlled trial	• Warm water immersion (42 °C) • Subjects were seated in a tank with water up to top-sternal level	• Pre-treatment (T1) • Post-treatment (T2)	• Healthy women (n = 9) • Aged 25 ± 5 years (mean) • No mention of calculation of sample size	30 min	24 times for 8 weeks	• Control (n = 9): cycling (70% HRmax)	• Brachial artery flow-mediated dilation • Cardiorespiratory fitness	• Two outcome variables improved after both warm water intervention and cycling • Passive heat training through warm water intervention can be a useful alternative to exercise training.	Thermal effect
Costello et al. [21]	Randomized crossover trial	• Cold water immersion (14 ± 1 °C) • Subjects were seated in tank with water up to umbilicus level		• Healthy men (n = 8) and women (n = 6) • Aged 21.9–25.1 years (mean) • No mention of calculation of sample size	30 min	Single	• Self-control (crossover) (n = 14): tepid water immersion (28 ± 1 °C)	• Knee joint position sense	• No significant difference between pre- and post-test for both cold and tepid water • Cold water immersion cannot reduce knee joint position sense	Thermal effect
Herrera et al. [22]	Quasi-experimental	• Cold water immersion (10 °C) • Subjects were seated in an acrylic container with water below the level of the head of the fibula	• Pre-treatment (T1) • Post-treatment (T2)	• Healthy men (n = 18) and women (n = 18) • Aged 20.5 ± 1.9 years (mean) • Sample size was calculated using Stata	15 min	Single	• Comparative 1 (n = 12): ice massage • Comparative 2 (n = 12): ice pack	• Skin temperature • Nerve conduction parameters	• Cold water immersion is the most effective modality for changing the nerve conduction parameter	Thermal effect
Hu et al. [23]	Randomized crossover trial	• Warm water immersion (41–43 °C) • Subjects were seated with legs and feet in a plastic bucket with the water level below the knees	• Pre-treatment (T1) • Post-treatment (T2)	• Healthy young (n = 16) and older women (n = 16) • Young women aged 25.4 ± 0.4 years (mean) and older women aged 59.8 ± 1.7 years (mean) • No mention of calculation of sample size	30 min	Single	• Self-control (crossover): sedentary seating in chairs	• Cardio-ankle vascular index indicated arterial stiffness • Tympanic temperature	• Main time effect (+) in both women • HR (↑), diastolic BP (↓) in young women • Tympanic temperature (↑) in both groups of women • Warm water immersion results in transient improvement of systemic arterial stiffness, mediated by elevation of core temperature • Repeated thermal therapy can promote cardiovascular health	Thermal effect
Wakabayashi et al. [24]	One group pre- and post-test	• Repeated mild cold water immersion (26 °C) • Subjects were seated in tank with water up to xiphoid level	• Pre-treatment (T1) • Post-treatment (T2)	• Healthy men (n = 7) • Aged 21.3 ± 0.8 years (mean) • No mention of calculation of sample size	60 min	12 times over 4 weeks	-	• Body temperature (11 regional skin temperatures) • Skin blood flow on the forearm • Metabolic heat production • Cold-induced vasodilation	• Main effect of pre- and post-test acclimation is observed in mean skin temperature • Skin blood flow was significantly lower post-test than pre-test • Index of cold-induced vasodilation was significantly lower in post-test acclimation than in pre-test acclimation • The repeated cold immersion in 26 °C water was sufficient to induce the insulative-type of cold adaptation.	Thermal effect
Brunt et al. [25]	Non-randomized trials	• Warm immersion up to the shoulder (40.5 °C) for 25–30 min and up to the waist for 60 min • Subjects stayed in hot tub until the rectal temperature reached 38.5 °C	• Pre-treatment (T1) • 1 week after treatment (T2) • 2 weeks after treatment (T3) • 4 weeks after treatment (T4) • 6 weeks after treatment (T5) • 8 weeks after treatment (T6)	• Healthy sedentary males (n = 4) and females (n = 6) • Aged 22.0 ± 1.0 years (mean) • No mention of calculation of sample size	90 min	36 times over 8 weeks	• Control (n = 10): thermo-neutral water immersion (36 °C)	• Carotid artery wall thickness and stiffness • Pulse wave velocity • Flow-mediated dilatation and post-occlusive reactive hyperemia • Endothelium-dependent dilatation	• Flow-mediated dilatation with passive heat therapy (warm immersion) was significantly elevated at 2, 6, and 8 weeks • Passive heat therapy significantly increased post-occlusive reactive hyperemia by 6 weeks • Passive heat therapy significantly reduced carotid artery wall thickness by 8 weeks • There was a significant main effect of time on the systolic blood pressure • Passive heat therapy using warm immersion results in increased endothelium-dependent dilatation and reduced arterial stiffness, wall thickness, and blood pressure.	Thermal effect
Streff et al. [26]	Randomized crossover trial	• Hot water immersion (47–48 °C) • Subjects were immersed in water up to the wrist in a 12-L tank	• Pre-treatment (T1) • Post-treatment with hot or cold water (T2) • Post-alternating treatment with hot and cold water (T3)	• Healthy men (n = 17) and women (n = 18) • Aged 24 years (median) • Sample size was calculated using G*power	75 min	Single	• Self-control (crossover): cold water immersion (3–4 °C)	• Subjective pain intensity, pain threshold, pain intolerance level using the VAS • Unpleasantness and affectivity • Physiological parameters (BP, HR, RR)	• Both pain thresholds and pain tolerance levels were significantly higher for cold water immersion than for hot water immersion • Hot water immersion produced a slightly higher subjective pain experience and was tolerated for a shorter period of time • BP was significantly higher in the cold water immersion trial • In cold water immersion, the HR parameters varied as the sympathetic activity was higher than that in hot water immersion	Thermal effect
Wijayanto et al. [27]	One group pre- and post-test	• Hot water immersion (38 °C, 40 °C, and 42 °C) • Subjects were immersed in water up to the knee level in a chamber	• Pre-treatment (T1) • 15 min after treatment (T2) • 30 min after treatment (T3) • 45 min after treatment (T4)	• Healthy men (n = 11) • Aged 22.1 ± 1.1 years (mean) • No mention of calculation of sample size	45 min	Single	-	• Short-term memory span • Rectal temperature • BP • Subjective thermal comfort and thermal sensation • Tissue oxygenation index in the pre-frontal cortex • Change in oxy-Hb level • Change in deoxy-Hb level	• Significant main effect of water temperature on change in the rectal temperature and HR after 45 min • Change in oxy-Hb increased in all three different conditions of water temperature (time effect): significantly higher in the 42 °C condition than in the 38 °C condition • Change in deoxy-Hb did not differ among the three conditions • Different temperature conditions induced little effect on cognitive functioning	Thermal effect
Kojima et al. [28]	Randomized crossover trial	• Hot water immersion (42 °C) • Subjects sat in a tank with water up to the neck	• Pre-treatment (T1) • Immediately after treatment (T2) • 15 min after treatment (T3) • 30 min after treatment (T4)	• Healthy men (n = 8) • Aged 25.4 ± 3.3 years (mean) • No mention of calculation of sample size	20 min	-	• Self-control (crossover): thermo-neutral water immersion (35 °C)	• Core temperature • Mean arterial pressure • Heart rate • Serum BDNF level • Serum S100β level • Plasma cortisol level • Plt count • Monocyte count	• Core temperature was significantly higher at T2 and T3 • BDNF level was higher at T2 and T3, and returned to the baseline at T4. • Cortisol level was lower at T2 and returned to pre-test level during the recovery period	Thermal effect
Valizadeh et al. [29]	Controlled single-blinded parallel trial	• Hot water immersion (41–42°C) • To place foot in plastic container at a height of 10 cm	• Pre-treat (T1) • Post-treat (T2)	• Healthy elderly subjects (n = 23) • Aged 67.69 ± 4.28 years (mean) • Sample size was calculated	20 min	42 times over 6 weeks	• Comparative 1 (n = 23): massage with olive oil • Control (n = 23): no treatment	• Quality of sleep and sleep patterns using PSQI instrument	• Foot bath was effective in all components except sleep efficiency and use of sleep medication • Foot bath caused 22% reduction in the prevalence of sleep disorders compared to 18% reduction with the comparative intervention	Thermal effect
Kim et al. [30]	Quasi-experimental	• Hot water immersion (40 °C) • Subjects placed their foot in a footbath machine to a height of 20 cm above the ankle	• Pre-treatment (T1) • 1 week after treatment (T2) • 2 weeks after treatment (T3) • 3 weeks after treatment (T4) • 4 week after treatment (T5)	• Healthy elderly subjects (n = 10) • Aged 81.6 ± 4.5 years (mean) • Sample size was calculated using G power • All groups (experimental, comparative, and control) were divided into the subgroups good-sleep and poor-sleep	30 min	28 times over 4 weeks	• Comparative 1 (n = 10): water immersion (36.5 °C) • Control (n = 10): no treatment	• Sleep patterns assessed using ATG machine (Mini-Mitter Co., Inc., Bend, OR, USA) • Sleep-disturbed behaviors were assessed using SDI instrument	• There were no significant differences in total sleep between groups and between measurement times • In the sleep effectiveness of the experimental group, there was a significant interaction between group and time	Thermal effect

HR: Heart Rate, BP: Blood Pressure, FFA: Free Fatty Acid, RBC: Red Blood Cell Count, WBC: white blood cell count, I/R: Ischemia-reperfusion (inflation and reperfusion using inflatable occlusion cuff), SDI: Sleep Disorders Inventory, FMD: Flow-mediated dilation, T-chol: total cholesterol, LDL-C: low-density lipoprotein, HDL-C: high-density lipoprotein, TG: triglyceride, CBC: complete blood count, Hb: hemoglobin, Ht: hematocrit, Plt: platelets, HRmax: maximum heart rate, RR: Respiratory Rate, VAS: Visual Analog Scale, PSQI: Pittsburgh Sleep Quality Index, BDNF: brain-derived neurotrophic factor.
